# Incentivising general practice: a review of the Scottish targeted enhanced recruitment scheme (TERS) through recruitment and training data

**DOI:** 10.3399/BJGPO.2024.0289

**Published:** 2025-09-10

**Authors:** Markus Chan, Peter D Donnelly, Frank Sullivan, Lindsey Pope, Nitin Gambhir

**Affiliations:** 1 Population and Behavioural Sciences Division, School of Medicine, University of St Andrews, St Andrews, UK; 2 School of Health and Wellbeing, University of Glasgow, Glasgow, UK; 3 NHS Education for Scotland, Glasgow, UK

**Keywords:** primary care workforce, primary care education, education, workforce planning, workforce, general practice

## Abstract

**Background:**

One of the challenges facing UK general practice is the dwindling workforce, particularly in deprived or remote areas. One solution is to increase trainees’ exposures to these environments by incentivising training in these locations. The Scottish Targeted Enhanced Recruitment Scheme (TERS) offered a one-time grant to general practice specialty trainees (GPSTs) in historically under-subscribed training programmes from 2017–2023.

**Aim:**

To evaluate the impact of the TERS grant on GPST recruitment across Scotland and within the targeted programmes.

**Design & setting:**

Analytical observational study of recruitment and training data from NHS Education for Scotland.

**Method:**

Recruitment data from 2015–2023 was provided by NES. Odds ratios (ORs) of recruitment rates were calculated before and after the introduction of TERS, as well as between programmes based on their eligibility for TERS.

**Results:**

The TERS recruited 734 individuals to GP specialty training posts across Scotland. In total, 1522 individuals were recruited to programmes not eligible for TERS in the same timeframe. The odds ratio of the effect of the TERS grant on recruitment across all programmes was 4.81 (95% confidence interval = 3.87 to 5.99). The OR for the TERS grant on recruitment within eligible programmes was 2.33 (95% CI = 1.74 to 3.12).

**Conclusion:**

We found a one-off £20 000 conditional grant was associated with a doubling of the odds of recruitment to historically under-filled GP specialty training programmes. Further work is needed to explore the effect of the TERS grant, and its withdrawal in 2024, on retention and the GP workforce.

## How this fits in

Previous research has emphasised the need for primary care-specific strategies to improve recruitment and retention, particularly in rural and underserved areas. This study aimed to evaluate the effectiveness of a targeted financial grant in improving recruitment of GP specialty trainees (GPST) in underserved areas of Scotland. We found this grant had a statistically significant effect (*P*<0.0001) on GPST recruitment in Scotland overall as well as within eligible programmes. This study highlights the potential of targeted financial incentives to help address current and future primary care workforce shortages.

## Introduction

General practice remains under significant strain owing to simultaneous challenges of performance pressure, limited funding, and falling morale within the workforce.^1^ One factor is the relatively low number of new GPs entering the workforce. Although recent recruitment cycles have achieved high fill rates, this has not always been the case. Importantly, national level figures may not reflect regional variation or less-than-full-time (LTFT) working patterns, which may influence future workforce planning.^2^

Decisions around specialty training pathways are complex, although multiple factors contribute to this complexity.^3,4^ Financial factors are important to highlight; for example, Scanlan *et al* found postgraduate doctors were willing to trade 45% of their expected earnings to train in a more desirable location.^3^ Conversely, other studies have found financial incentives nonetheless have some effect in encouraging trainees to train in less desirable locations.^5–7^ This study adds empirical data to this area of research.

The Scottish Targeted Enhanced Recruitment Scheme (TERS) aimed to improve recruitment to general practice specialty training programmes through a one-off grant of £20 000 to GP specialty trainees (GPSTs) who train in historically underserved training programmes.^8^ Eligible programmes included: the Dumfries and Galloway, Ayrshire and Arran, Lanarkshire programmes from the West of Scotland deanery; the Eastward and Westward programmes in the East of Scotland deanery; and the Caledonian and Rural Track programmes from the North of Scotland deanery. For the latest cohort (2023–2024), funding was reduced from £2.4m to £1.0m, and the eligible programmes were restricted to four programmes: the Borders (South-East of Scotland deanery), Caledonian, Rural Track, and Dumfries and Galloway programmes.^8^

Funding was provided by the Scottish Government as part of the National Health and Social Care Workforce Plan. The plan also included schemes such as the ’Golden Hello’ to support recruiting qualified GPs to practices in areas of deprivation and/or rural and remote practice.^9,10^

### What is currently known about the TERS?

In their 2019 survey, Lee and Cunningham found TERS influenced an important minority (19%–21%) of responders in choosing a Scottish training programme.^11^ However, their survey assessed a single recruitment cycle and the longer-term impact of TERS is unclear.

### Purpose and aims of this study

This project assessed the impact of the TERS grant on the recruitment of GPSTs between programmes within Scotland. This project also assessed how recipients of the TERS grant compared with other GPSTs and the wider primary care workforce.

The specific research aims were as follows:

1. to quantify the impact of TERS on GPST recruitment across eligible training programmes;
2. to quantify the impact of TERS on GP training recruitment in Scotland in general;
3. to explore how recipients of TERS grants compare with other GPSTs; and
4. to explore how recipients of TERS grants compare with the rest of the UK general practice workforce.

## Method

### Population and eligibility

All applicants who were successfully enrolled on eligible training programmes automatically received the TERS grant at the start of training with no restrictions on age or prior training. Individuals leaving training early for any reason were required to repay this proportionately to the outstanding training time; for example, a trainee who left training after 2 years of a 3-year programme would have to pay back one-third of the grant.

### Data sources

NHS Education for Scotland (NES) provided data on the number of available posts and recruited GPSTs by programme per year from 2015–2023 from their employment records. This dataset included data from 3 years before the implementation of the TERS grant (2015–2017) to provide robust comparison. The dataset also contained self-reported information from the recipients of the TERS grants, which included place of training, gender, age group, and exit outcome. Notably, data were incomplete regarding the reasons for leaving training and post-training work activity.

Workforce and population-level demographic data were imported from publicly available datasets created by the UK Government, the Scottish Government, and NES.^12,13^

### Data analysis

Data were exported to RStudio (version 4.0 ’Ocean Storm’, using R 4.3.3) for cleaning and analysis using the code libraries tidyverse, readxl, and rvest. Recruitment rates were calculated as a percentage of total available places. No unexpected results or data cleaning errors were identified in the analysis. Comparisons were made between and within programmes by TERS eligibility and corrected for overall trends in GPST recruitment.

### Outcomes

The primary outcome of interest was the effect of the TERS grant on the recruitment of GPSTs to historically under-subscribed programmes. The effect size was expressed as an odds ratio (OR) of filled posts to unfilled posts.

Secondary outcomes of interest were as follows: the OR comparing recruitment between programmes after the introduction of the TERS grant; and the variance between the TERS grant recipients and the Scottish and national GP workforce.

## Results

### Recruitment of GPSTs to eligible programmes

**Figure 1.**
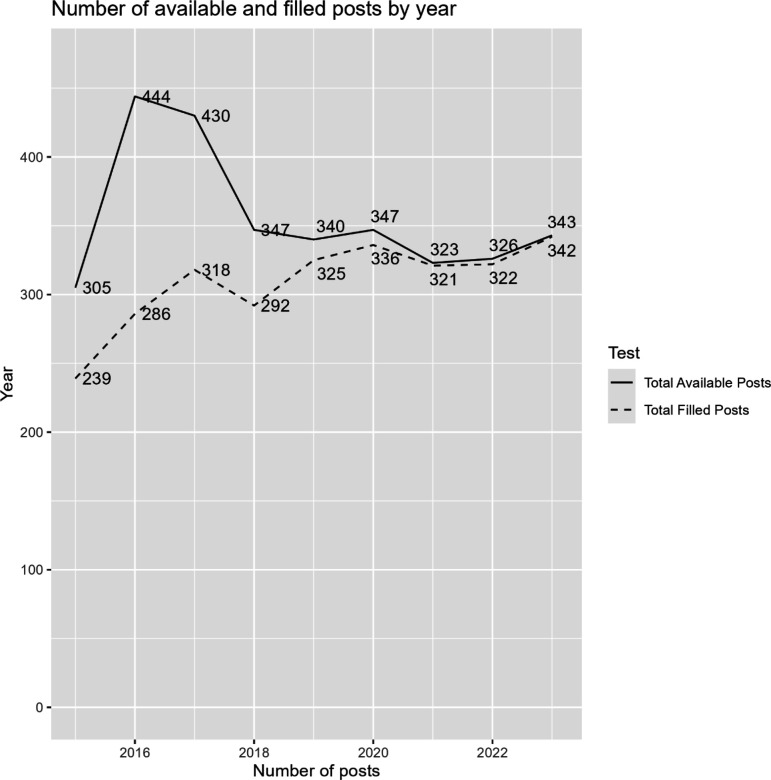
Total number of posts available and filled by year

From 2017–2023, the scheme recruited 734 individuals across Scotland. In total, 1522 individuals were recruited to programmes not eligible for TERS in the same timeframe. As seen in Figure 1, there were 2 years (2016 and 2017) where there were more available posts, although this was otherwise stable at approximately 350 posts per year. This variance was owing to a Scottish Government scheme where 100 new GPST posts were created to increase the number of GPs.^14–16^ Average recruitment increased in eligible programmes from 84.5 per year to 123.9 per year: this was not statistically significant (*P* = 0.24).

Average recruitment rate within eligible programmes increased from 57.3% before 2017 to 87.6% after the introduction of the TERS grant, with an OR of 2.33 (95% confidence interval CI = 1.74 to 3.12). This effect was more pronounced in the first cohort of TERS (2017–2022 inclusive), where the effect size of the grant was 2.43 (95% CI = 1.80 to 3.30). Although there was only 1 year of data for the newest cohort of TERS (2023 onward), the grant appears to still be effective. Importantly, the recruitment rate in 2023 for programmes that were previously eligible for TERS grants remained stable at 99.3%.

### Impact of TERS on GP recruitment overall

The overall OR of the effect of the TERS grant on recruitment across all programmes was 4.81 (95% CI = 3.87 to 5.99). Recruitment to GP specialty training increased across the study period. As seen in Table 1, recruitment across all programmes increased from 78.4% in 2015 to 99.7% in 2023. Across programmes not eligible for TERS, recruitment increased from 90.5% in 2015 to 100% in 2023. There was a non-significant increase in recruitment across all programmes in 2017.

**Table 1.** Recruitment rates (%) by TERS eligibility by year

Year	Overall	Programmes eligible for TERS	Programmes not eligible for TERS
Before implementation of TERS	70.1%	57.3%	78.4%
2015	78.4%	58.6%	90.5%
2016	64.4%	56.4%	69.8%
After implementation of TERS	91.9%	87.6%	94.7%
2017	74.0%	63.6%	80.9%
2018	84.1%	70.7%	95.3%
2019	95.6%	95.5%	95.7%
2020	96.8%	97.1%	96.7%
2021	99.4%	99.2%	99.5%
2022	98.8%	98.5%	99.0%
2023	99.7%	99.3%	100.0%

TERS = Targeted Enhanced Recruitment Scheme

**Figure 2.**
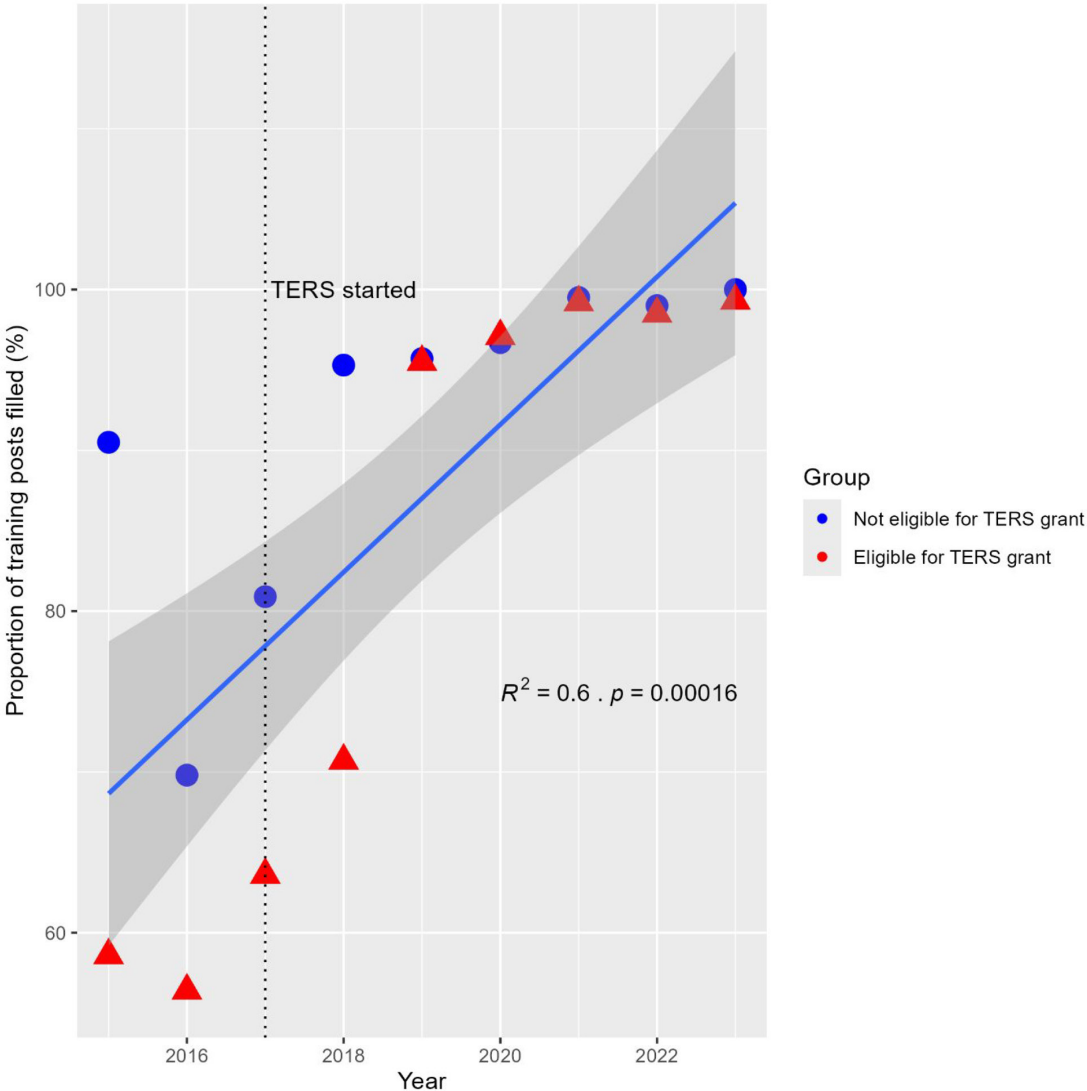
Linear regression model of proportion of training posts filled by TERS eligibility by year. TERS = Targeted Enhanced Recruitment Scheme

The general trend in Scottish GP specialty training recruitment over time was modelled using linear regression (Figure 2). This accounts for much of the observed variance (*R* ^*2*^ = 0.60). Comparatively, the TERS grant accounted for a smaller proportion of observed variance (*R* ^*2*^ = 0.10).

### Demographics of TERS grant recipients

As seen below (Table 2), when compared with the Scottish general practice workforce and the overall UK workforce, TERS recipients were more likely to be from a non-White ethnic minority group (OR 2.41, 95% CI = 1.26 to 4.70, *P*<0.01, and OR 2.16, 95% CI = 1.14 to 4.16, *P* = 0.02, respectively).

**Table 2.** Demographics of TERS grant recipients versus Scottish general practice workforce versus UK general practice workforce versus UK overall workforce

Ethnicity	TERS grant recipients	Scottish general practice workforce	UK general practice workforce	UK overall workforce
White	57.6%	76.6%	51.3%	75.1%
Non-White	42.4%	23.4%*	48.7%	24.9%*
Asian	14.2%	10.3%	27.7%	10.4%
Black	10.9%	3.2%	9.2%	6.0%
Mixed	3.0%	1.6%	2.1%	1.7%
Other	4.9%	1.2%	3.1%	2.3%
Unknown	9.4%	7.1%	6.6%	4.5%

TERS = Targeted Enhanced Recruitment Scheme

*The original datapoints reported were 0.1% off due to rounding within the sub-groups. This has been corrected here using the underlying data reported.

Approximately 62% of TERS grant recipients had a British primary medical qualification (PMQ). This is comparable with the wider UK, where 57% of GPSTs hold a British PMQ.^17^ Compared with other Scottish GPSTs, a significantly larger proportion of TERS recipients held a British PMQ (28.9% versus 38%, OR 1.50, *P*<0.05).

### Training outcomes

Regarding training duration, 76.8% of TERS recipients were in full-time training. Of those working less-than-full-time (LTFT), most were working at 0.8 full-time equivalence (14.6%). Comparatively, more than one-third of GPSTs are currently working or plan to work LTFT.^18^ While, 36.0% of TERS recipients have successfully completed training. Approximately 5.9% of recipients left training.

## Discussion

### Summary

This study demonstrates the effectiveness of a financial incentive to improve recruitment. In this study, a one-off £20 000 conditional grant was associated with a doubling of the odds of recruitment to historically under-filled GP specialty training programmes. The size of the grant is worth highlighting: the £20 000 offered by the TERS grant represents an approximate 15% increase in salary over the programme.^19^ However, the effect of this grant on trainee numbers was less clear; the average number of trainees per year increased by 47%, although we may have been underpowered to meet statistical significance.

Some of the observed changes likely represent larger trends in national recruitment. General practice specialty training and medical specialty training in general in the UK is becoming more competitive.^20,21^ Changes to GP recruitment are likely to underlie some of this; for example, the application process is now centralised, which may make applying to multiple programmes easier for potential GPSTs. Similarly, recent changes have allowed international medical graduates (IMGs) to apply directly to general practice specialty training, resulting in more IMGs in training and in practice.^17,21–23^

This study found recipients of the grant were more representative of the UK rather than Scottish workforce.^17^ This suggests the scheme attracted applicants beyond Scotland, although more data is needed to confirm this assertion. Furthermore, IMGs have been incentivised to join Scottish GP training programmes through initiatives such as the Scottish Trainee Enhanced Programme which provides additional support to IMGs in Scotland.^24^ Importantly, it is unclear whether these changes lead to increased full-time-equivalent numbers on the GP register.

### Strengths and limitations

This study adds to the growing body of evidence of interventions to improve recruitment using a robust, multi-year, national dataset. The availability of data from programmes not eligible for the TERS grant as well as historic data enables robust comparison and helps empower the statistical analyses within the study.

Our analysis creates a clear reference point for other national recruitment schemes. Notably, the geographical distribution of the targeted training programmes enables comparisons with schemes aimed at improving recruitment to semi-rural and rural general practice.

This study also demonstrates the effectiveness of routinely collected data. The dataset supplied by NES was derived from routinely available recruitment data, yet allowed detailed analyses of the impact of the TERS grant on recruitment.

A significant limitation of this study is the limited participant data. This study cannot therefore comment on issues such as the impact of the grant on applications to Scottish GP specialty training, or the relative experiences of grant recipients in GP specialty training, examination results and completion of training. Although the data allow some conjecture to be made regarding the types of applicants and the reasons the grant may have been effective, further work to qualify this would be beneficial.

A further limitation is the variable data in the lead-in period. In the 2 years before the TERS grant was introduced, the number of available places for GP specialty training was higher than in the years the scheme was active owing to a government scheme that created new training posts. Having more historic data would allow this variance to be better corrected for, resulting in more accurate analyses. Similarly, more data is needed to formally assess how changing which programmes are eligible for the TERS grant has affected its impact.

It is also not yet possible to comment on the effect of the TERS grant on retention. Preliminary data from NES suggests many TERS grant recipients are still registered to work within their training health board; however, it is unclear whether they are working within underserved communities. Evidence from Australia suggests financial incentives may help recruit GPs, but may be less effective at retaining existing staff.^25,26^ Further research is needed to see whether this holds true in the UK.

### Comparison with existing literature

The findings of this study align with multiple systematic reviews that show financial incentives may significantly influence decisions around place of training.^6,7,27^ There is limited evidence exploring the role of the size of a financial incentive and its effect on medical recruitment, although evidence from other fields and contexts confirms the intuition that larger financial incentives are more effective than smaller incentives.^28–31^

However, the effect of the TERS grant in increasing the number of trainees was less clear; the average number of trainees per year increased by 47%, although we may have been underpowered to meet statistical significance. This is especially true when considering the wider context of larger trends in GP specialty training recruitment, with increased numbers of GPSTs not necessarily equating to increasing numbers of GPs. Approximately 1 in 20 recipients of the TERS grant in this study left GP specialty training. This may be owing to how data were recorded, although this finding generally aligns with recent trends seen across postgraduate doctors.^32–34^ Recent reports also suggest GPSTs are facing increasing rates of burnout; worryingly, the number of recently qualified GPs leaving the profession continues to rise.^35–38^

### Implications for research and practice

Further work is needed to support the implementation and diffusion of evidence-based interventions like TERS.^39^ There is a need for qualitative research to explore how trainees experienced programmes like this one. Similarly, further work assessing how the magnitude of the incentive relates to its effect would be valuable for policymakers to ensure efficient use of resources. Importantly, increased recruitment of GPSTs may not necessarily lead to increased numbers of GPs. Further research investigating recipients’ experiences of training and post-training outcomes would help clarify the effect of the TERS grant on GP retention. To this end, collecting exit data from TERS grant recipients on completion of training may be helpful.

In conclusion, this study adds to the growing body of evidence showing financial incentives can improve recruitment. Evidence-based interventions to improve recruitment are especially relevant in the current climate of general practice.^40,41^ Although general trends in postgraduate specialty training may account for most of the observed change, the TERS grant appears to improve recruitment to historically less popular GP specialty training programmes in Scotland. Recipients of the TERS grant reflected the UK-wide workforce, suggesting the grant was successful in attracting non-Scottish doctors to Scottish general practice training programmes. The Scottish targeted enhanced recruitment scheme was discontinued following a marked increase in the number of applications for available training posts during the intervening period. In addition, evolving fiscal pressures and shifting organisational priorities contributed to the decision to withdraw the scheme.

## Data

The dataset relied on in this article is available from the corresponding author (FS) on reasonable request.
